# The impact of anemia within 24 h after surgery on the prognosis of patients with pseudomyxoma peritonei

**DOI:** 10.3389/fonc.2025.1487823

**Published:** 2025-11-18

**Authors:** Xiaoyun Gao, Hang Guan, Yiyao Li, Liduo Kou, Hua Tian, Junhui Jia, Wei Bai, Yu Bai, Yanhui Di, Ruiqing Ma, Xinhua Wang

**Affiliations:** 1 Department of Blood Transfusion, Aerospace Center Hospital, Beijing, China; 2 Department of Mucinous Tumor, Aerospace Center Hospital, Beijing, China

**Keywords:** pseudomyxoma peritonei, anemia, hemoglobin, prognosis, overall survival

## Abstract

In this study, we examined the effect of postoperative anemia on the prognosis of 721 patients with pseudomyxoma peritonei (PMP). Based on the initial hemoglobin (Hb) levels measured after surgery, the patients were categorized into the normal Hb (n = 65) and anemia groups (n = 656 patients, 91%), and the anemic patients were further divided into four subgroups based on their Hb levels. Patients with normal postoperative Hb levels had a significantly higher 5-year survival rate (80%) than those with anemia (67.4%). In addition, patients with Hb levels of 81–90 g/L had the shortest median survival duration of 36.3 ± 1.4 months. Tumor recurrence rates were consistent across the groups, whereas plasma and red blood cell transfusion volumes differed among the anemia subgroups. The postoperative cytoreduction rate, peritoneal carcinomatosis index, tumor grade, and Hb level were identified as factors significantly affecting postoperative survival. In conclusion, anemia developing within 24 h after surgery significantly affects the 5-year survival rate in patients with PMP, and the severity of anemia is a crucial risk factor.

## 1 Introduction

Pseudomyxoma peritonei (PMP) is a rare clinical syndrome characterized by the widespread intraperitoneal dissemination of mucinous tumor cells, leading to the accumulation of large amounts of mucinous or gelatinous ascites within the peritoneal cavity (1–5). The tumor cells involved in PMP originate from nearly all abdominal organs including the ovaries, rectum, pancreas, and colon (6–11). However, the most common source of PMP is perforated appendiceal mucinous tumors, which account for approximately 87.2%–94% of all cases (12–15). The exact incidence rate of PMP remains to be determined. Some studies have reported an incidence of 1–2 per million (7, 16), whereas others estimated an incidence of 3–4 per million cases per year (17–19). The current standard treatment for PMP is complete cytoreductive surgery (CRS), which involves organ resection and peritonectomy, combined with hyperthermic intraperitoneal chemotherapy (HIPEC) (20, 21). Given the complexity and duration of CRS+HIPEC, involvement of multiple organs, and extensive intraoperative blood loss, patients might develop or experience worsening anemia following the procedure. Relatively little is known regarding the association between postoperative anemia and prognosis in patients with PMP. Studies show that preoperative anemia can adversely affect organ perfusion, postoperative wound healing and the overall recovery of patients (22–24). Furthermore, anemia is associated with increased postoperative complications, decreased response to chemotherapy, and worse outcomes in colorectal cancer patients (25, 26), and has been shown to increase the risk of short- and long-term mortality in patients with acute coronary syndrome (27–30). Although patients with PMP have a high risk of anemia before and after surgery due to the large amount of mucous ascites and complex surgical treatment, no study has reported a correlation between anemia and patient prognosis. In this study, we analyzed the incidence of anemia in PMP patients within 24 h after surgery and assessed its prognostic relevance.

## 2 Materials and methods

### 2.1 Case selection

PMP patients who were treated at the Mucinous Tumor Department of the Aerospace Center Hospital from January 2015 to December 2021 were retrospectively reviewed. The patients had been diagnosed according to the 2016 PSOGI consensus for PMP and received standard CRS+HIPEC. The exclusion criteria were as follows: 1) first postoperative test for hemoglobin (Hb) conducted more than 24 h after surgery, and 2) incomplete clinical data.

### 2.2 Assessment of anemia

Patients were categorized into normal Hb and anemia groups based on the results of the first Hb test conducted within 24 h after surgery. The criteria for anemia in men and women were Hb level <130 and <120 g/L respectively. Based on their Hb levels, patients with anemia were further stratified into the following subgroups: Hb ≤ 80 g/L, Hb = 81–90 g/L, Hb = 91–100 g/L, and Hb = 101–110 g/L.

### 2.3 Blood component transfusion

Transfused blood components were supplied by the Beijing Red Cross Blood Center. Red blood cells (RBCs) were either suspended or leuko-reduced in 1 U/2 U packets. Transfusions were ABO- and Rh-compatible, which was verified by cross-matching. All transfused components were irradiated with 25–30 Gy gamma radiation. In addition, ABO- and RhD-compatible frozen plasma samples (100 mL/200 mL) were obtained and thawed at 37°C for 25 min prior to transfusion.

### 2.4 Data collection

The following data were collected: sex, age, BMI (<18.5, 18.5–24, 24–28, >28), prior surgical score (PSS; 0–1 vs. 2–3), history of chemotherapy, cardiac function, history of hypertension, number of organs resected, postoperative cytoreduction rate (CCR; 0–1 vs. 2–4), peritoneal carcinomatosis index (PCI; ≤ 20 vs. > 20), tumor grade (1–2 vs. 3–4), postoperative recurrence status, recurrence-free interval (months), postoperative survival status and duration (months), postoperative transfusion volumes of RBCs (U), plasma (mL), and platelets (treatment dose), and the pre- and postoperative Hb levels (g/L) and platelet counts (1 × 10^9^/L).

### 2.5 Outcome evaluation

The primary endpoint was 5-year overall survival (OS). Secondary endpoints included tumor recurrence during the follow-up period and the postoperative transfusion volumes of RBCs (U), plasma (mL), and platelets (treatment dose). Cox multivariate regression was used to identify independent risk factors for postoperative OS and tumor recurrence.

### 2.6 Statistical analysis

Continuous variables were presented as the mean ± standard deviation, and the different groups were compared by independent t-tests. Categorical variables were presented as absolute numbers and proportions, and the data were compared between the groups using the chi- squared test. The survival and recurrence rates were calculated by the Kaplan–Meier method and compared between the different groups and subgroups using the log-rank test. Univariate and multivariate Cox regression models were established to identify the independent risk factors for postoperative OS and tumor recurrence. BMI, CCR, PCI, tumor grade, preoperative Hb levels, PSS, and history of chemotherapy were selected as covariates to assess the effects of anemia and its severity on survival. SPSS 26.0 was used for all statistical analysis, and P < 0.05 was considered statistically significant.

## 3 Results

### 3.1 Baseline characteristics

There were 721 patients in the study cohort, including 264 men and 457 women with ages ranging from 19–85 years (mean age, 57.3 ± 10.8 years). Based on the initial postoperative Hb test, 65 patients were categorized into the normal Hb group, and 656 patients were classified as anemic. Accordingly, the incidence of 24-h postoperative anemia was 91%. Patients with anemia were further divided according to the severity of anemia, including 34 patients (5.2%) with severe anemia (Hb levels ≤ 80 g/L), 115 patients (17.5%) with moderate anemia (Hb levels = 81–90 g/L), 203 patients (30.9%) with mild/moderate anemia (Hb levels = 91–100 g/L), and 304 patients (46.3%) with mild anemia (Hb levels = 101–130 g/L). The demographic data (gender, age, BMI), medical history (chemotherapy history, heart disease history, hypertension history), operation information (PSS, CCR, PCI, tumor grade, number of organs resected, operation time, intraoperative bleeding) and preoperative HB levels of the normal HB group and anemia group were compared. As shown in **Table 1**, there were significant differences in gender distribution, CCR, PCI, tumor grade, number of organs resected, preoperative HB levels, intraoperative bleeding and operation time between the two groups (P < 0.05).

**Table 1:** Comparative analysis of basic information between normal HB and anemia group.

Variable	Normal Hb	Anemia				*P* value
		<80	81-90	91-100	>100	
Gender						0.000*
Female	42	24	84	145	162	
Male	23	10	31	58	142	
Age	54.66 ± 12.77	60.94 ± 9.47	58.27 ± 10.41	57.24 ± 10.35	57.12 ± 10.84	0.067
BMI						0.091
<18.5	4	6	12	19	13	
18.5-24	33	16	67	95	158	
24-28	21	10	26	63	90	
>28	7	2	10	26	43	
PSS						0.155
0-1	35	14	54	95	170	
2-3	30	20	61	108	134	
Chemotherapy history						0.072
No	53	23	76	157	234	
Yes	12	11	39	46	70	
Cardiac disease history						0.828
No	62	33	112	199	294	
Yes	3	1	3	4	10	
Hypertension history						0.159
No	50	23	90	170	232	
Yes	15	11	25	33	72	
CCR						0.000*
0-1	39	1	24	55	112	
2-4	26	33	91	148	192	
PCI						0.000*
≤20	34	1	19	37	88	
>20	31	33	96	166	216	
Tumor grade						0.044*
1-2	56	30	106	188	257	
3-4	65	34	115	203	304	
Organectomy	3.32 ± 2.04	5.06 ± 3.00	3.61 ± 2.52	3.80 ± 2.28	3.37 ± 1.93	0.000*
Preoperative HB	130.78 ± 19.41	95.26 ± 12.86	101.70 ± 16.26	107.04 ± 15.06	117.68 ± 16.15	0.000*
Preoperative PLT	235.63 ± 77.32	294.71 ± 96.99	292.73 ± 111.55	282.70 ± 99.93	255.42 ± 90.05	0.000*
Intraoperative bleeding	1003.08 ± 865.2	2137.35 ± 1508.15	1612.61 ± 1194.65	1535.49 ± 1516.76	1300.82 ± 972.70	0.000*
Surgery time	447.82 ± 110.47	512.18 ± 150.51	498.62 ± 145.04	511.51 ± 132.43	478.20 ± 128.00	0.003*

*P < 0.05; BMI, body mass index; PSS, prior surgical score; CCR, cytoreduction rate; PCI, peritoneal carcinomatosis index.

### 3.2 Association between anemia and postoperative survival

**Figure 1:**
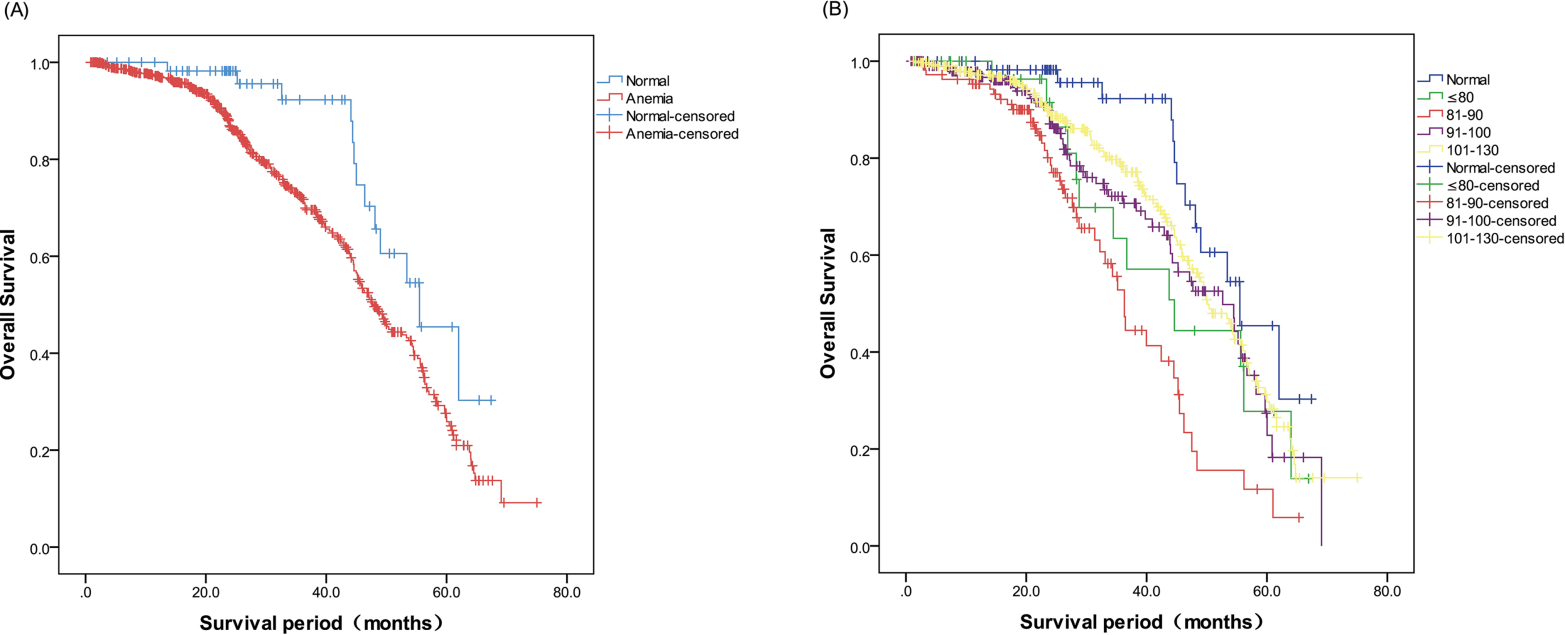
**(A)** Kaplan–Meier curves presenting postoperative overall survival (OS) in the normal and anemia groups. **(B)** Kaplan–Meier curves presenting postoperative OS in the anemia subgroups.

All patients enrolled in the study were successfully followed-up, and the median follow-up duration was 26 months (range: 2–76 months). Thirteen patients with normal Hb levels died during the follow-up period, and the median postoperative OS duration of this group was 55.5 months (range: 45.2–65.8 months). In addition, there were 214 deaths in the anemia group, and the median postoperative OS duration was 47.8 months (range: 44.8–50.8 months). As shown in **Figure 1A**, the 5-year postoperative survival rate was 80% in the normal Hb group, compared to only 67.4% in the anemia group, and the difference was significant (χ2 = 6.237, P=0.013). The mean OS durations in patients with severe, moderate, mild/moderate, and mild anemia were 44.6 ± 7.6, 36.3 ± 1.4, 52.6 ± 4.8, and 50.1 ± 2.3 months respectively. Furthermore, the OS in all anemia subgroups was significantly shorter than that in the normal group (χ2 = 27.784, P < 0.001), and patients with Hb levels of 81–90 g/L had the shortest median survival (**Figure 1B**).

### 3.3 Association between anemia and postoperative tumor recurrence

**Figure 2:**
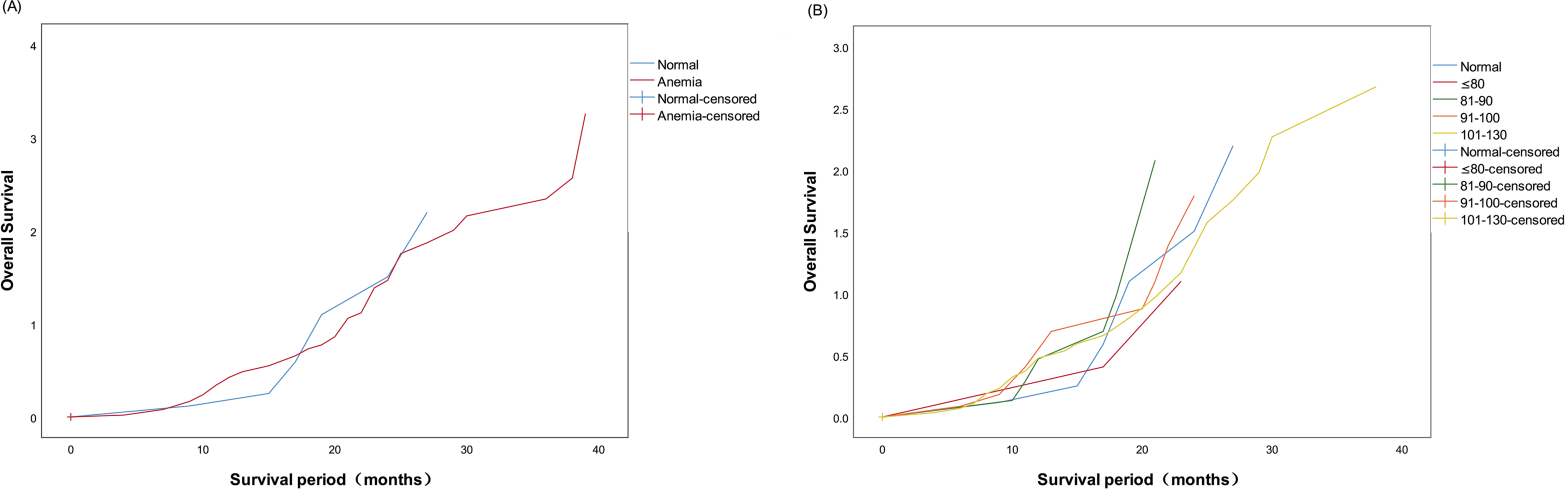
**(A)** Kaplan–Meier curves presenting the cumulative tumor recurrence rate in the normal and anemia groups after surgery. **(B)** Kaplan–Meier curves presenting the cumulative tumor recurrence rate in the anemia subgroups after surgery.

During the follow-up period, nine patients with normal Hb levels and 52 patients with anemia experienced tumor recurrence. No significant difference was observed in the 5-year recurrence rates (χ2 = 1.577, P=0.212) or the median time to recurrence (19.7 ± 2.1 vs. 23.1 ± 1.3 months) between the two groups. The cumulative postoperative tumor recurrence rates in the two groups are shown in **Figure 2**.

### 3.4 Factors affecting postoperative overall survival

The univariate Cox regression model revealed that BMI < 18.5 kg/m2, PSS 2–3, history of chemotherapy, postoperative CCR2–4, PCI > 20, tumor grade 3–4, preoperative Hb levels, and postoperative Hb levels of 81–90 and 91–100 g/L were significantly associated with postoperative OS in patients with PMP (all P < 0.05, **Table 2**). Furthermore, multivariate Cox regression analysis identified postoperative CCR2–4, PCI > 20, and tumor grade 3–4 as the independent risk factors for postoperative OS in the normal Hb group and all anemia subgroups (all P < 0.05, **Table 3**). The postoperative Hb level was identified as an independent risk factor for postoperative OS when comparing the four anemia subgroups to the normal Hb group (P=0.012, **Table 3**).

**Table 2:** Univariable cox regression analysis of the factors affecting postoperative survival.

Variable		HR	95.0% CI		*P*
Gender	Male vs. Female	1.03	0.79	1.34	0.849
Gge	Every 1 year	1.01	1.00	1.02	0.196
BMI					0.043*
18.5-24	vs <18.5	1.67	0.92	3.02	0.091
24-28	vs <18.5	1.15	0.73	1.83	0.546
>28	vs <18.5	0.84	0.51	1.40	0.504
PSS	0–1 vs 2-3	1.46	1.12	1.90	0.005*
Chemotherapy history	Yes vs No	1.50	1.15	1.97	0.003*
Cardiac disease history	Yes vs No	1.01	0.52	1.97	0.975
Hypertension history	Yes vs No	1.04	0.77	1.40	0.821
Organectomy	n	1.04	0.98	1.11	0.199
CCR	0–1 vs 2-4	2.89	1.99	4.20	<0.001*
PCI	≤20 vs >20	3.55	2.27	5.57	<0.001*
Tumor grade	1–2 vs 3-4	1.76	1.27	2.45	0.001*
Preoperative HB	g/L	0.98	0.98	0.99	<0.001*
Postoperative HB binary grouping	Anemia vs Normal	2.01	1.15	3.53	0.014*
Postoperative HB five groups					<0.001*
≤80	vs Normal	2.03	0.94	4.39	0.071
81-90	vs Normal	3.83	2.05	7.15	<0.001*
91-100	vs Normal	1.97	1.07	3.61	0.029*
101-130	vs Normal	1.71	0.961	3.044	0.068

*P < 0.05; BMI, body mass index; PSS, prior surgical score; CCR, cytoreduction rate; PCI, peritoneal carcinomatosis index.

**Table 3:** Multivariate cox regression analysis of the factors affecting postoperative survival.

Variable		Two groups				Five groups			
		HR	95.0% CI		*P*	HR	95.0% CI		*P*
BMI									
<18.5					0.489				0.571
18.5-24	vs <18.5	1.05	0.57	1.95	0.868	0.98	0.53	1.83	0.960
24-28	vs <18.5	0.96	0.60	1.53	0.860	0.93	0.58	1.49	0.759
>28	vs <18.5	0.77	0.46	1.28	0.310	0.76	0.45	1.27	0.292
CCR	0–1 vs 2-4	1.77	1.11	2.82	0.014*	1.77	1.11	2.82	0.016*
PCI	≤20 vs >20	2.30	1.34	3.93	0.002*	2.34	1.36	4.03	0.002*
Tumor grade	1–2 vs 3-4	2.33	1.63	3.31	0.001*	2.35	1.66	3.34	0.001*
Preoperative HB	g/L	0.99	0.98	1.00	0.122	1.00	0.99	1.01	0.521
PSS	0–1 vs 2-3	1.15	0.86	1.55	0.340	1.14	0.85	1.53	0.389
Chemotherapy history	Yes vs No	1.12	0.83	1.52	0.469	1.14	0.84	1.55	0.396
Postoperative HB binary grouping	Anemia vs Normal	1.34	0.75	2.39	0.330	–	–	–	–
Postoperative HB five groups									0.012*
≤80	vs Normal	–	–	–	–	1.15	0.50	2.63	0.739
81-90	vs Normal	–	–	–	–	2.40	1.22	4.73	0.011*
91-100	vs Normal	–	–	–	–	1.40	0.74	2.65	0.304
101-130	vs Normal	–	–	–	–	1.25	0.69	2.27	0.454

*P<0.05; -, blank.

### 3.5 Postoperative transfusion volumes for RBCs, plasma, and platelets

**Figure 3:**
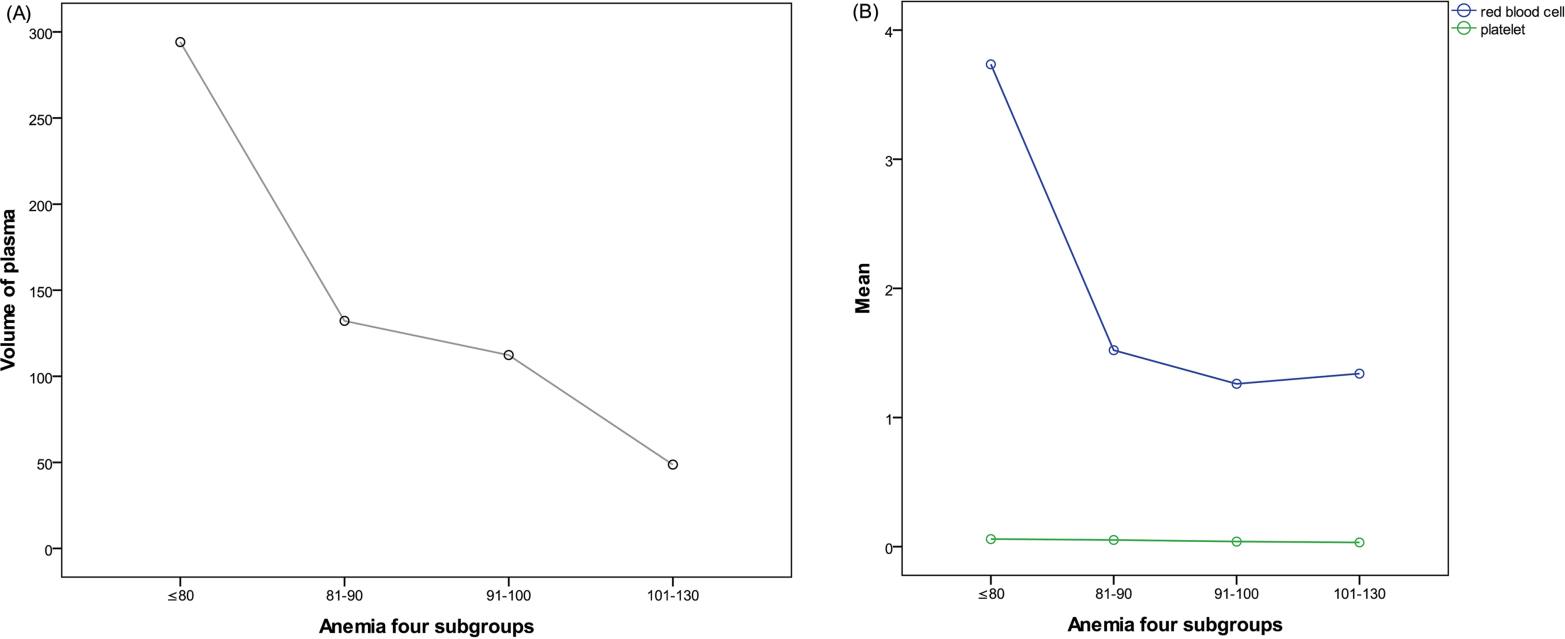
**(A)** Postoperative plasma use in patients with anemia; **(B)** Postoperative use of red blood cells and platelets in patients with anemia.

The anemic patients received postoperative transfusions of blood components depending on hemorrhaging, oxygen deficiency, and other symptoms, the results of Hb and platelet testing, and coagulative function. RBC infusion was given when the Hb level was less than 70g/L, platelet transfusion was performed when the cell count dropped below 20×10^9^/L, and plasma transfusion was given to patients with abnormal coagulation function. There were significant differences in the volumes of plasma and RBC transfusions among the four anemia subgroups (all P < 0.05), whereas that of platelet transfusion was similar across all subgroups (F=0.107, P=0.956). The results have been summarized in **Table 4** and **Figure 3**.

**Table 4:** Postoperative blood component infusion in patients with anemia.

Group	Plasma(mL)	Red blood cell (U)	Platelet (U)
≤80	294.12 ± 378.95	3.74 ± 3.38	0.06 ± 0.24
81-90	132.17 ± 324.05	1.52 ± 2.18	0.05 ± 0.03
91-100	112.32 ± 250.93	1.26 ± 2.48	0.04 ± 0.43
100-120/130	48.68 ± 180.94	1.34 ± 3.05	0.03 ± 0.24
F	15.26	8.66	0.11
*P*	0.001*	0.001*	0.956

*P<0.05.

## 4 Discussion

PMP is a rare malignancy of the peritoneal surface and is characterized as a chronic and progressively advancing disease. The current gold standard for treatment is a combination of CRS and HIPEC (20, 21). The 9th International Congress on Peritoneal Surface Malignancies endorsed CRS+HIPEC as the standard treatment for PMP in 2014 (31). The combination of CRS and HIPEC has been demonstrated to either cure patients or palliatively reduce tumor size, thereby extending survival and alleviating symptoms (32, 33). Ansari et al. reported 5- and 10-year OS rates of 87.4% and 70.3% respectively in 1000 patients with appendiceal tumors following CRS+HIPEC (34). Likewise, an Australian single-center cohort study reported median OS of 104 months and a 5-year survival rate of 75% among these patients (35). Consistent with previous reports, we found that the 5-year OS rate in patients with normal Hb levels within 24h of undergoing CRS+HIPEC was 80%.

Anemia is a frequent complication of cancer progression and treatment. The incidence of pretreatment anemia in cancer patients is approximately 40%, and may increase to 50%–90% after treatment (36–38). Nearly one-third of patients exhibit anemia prior to elective surgery, and approximately 50% of these cases are the result of deficiencies in iron, folic acid, and vitamin B12 (39). Furthermore, surgical trauma resulting in overt and occult blood loss can exacerbate postoperative anemia. In fact, the incidence rate of anemia following hip and knee joint replacement surgeries exceeds 80% (36), and approximately 84.6%–88.5% of patients undergoing hip fracture surgeries (39), and up to 89.2% of those undergoing bone tumor surgeries (40) develop postoperative anemia. PMP is a slow-progressing cancer with non-specific clinical manifestations, which often leads to late-stage diagnoses and subsequent anemia in many patients. Furthermore, CRS typically involves extensive peritoneal resections, often requiring complex postoperative reconstruction that results in significant blood loss (41). These factors contribute significantly to the high rate of postoperative anemia in patients with PMP. In this study, 91% of patients with PMP developed anemia within 24h of undergoing CRS+HIPEC, and the most common presentation was mild anemia. The causes of anemia in patients with PMP are complex and have not been clearly defined. We found that female patients were more likely to develop postoperative anemia. Other factors that were related to postoperative anemia included preoperative HB level, CCR, PCI, tumor grade, number of organs removed, intraoperative blood loss and length of operation. Patients with CCR, PCI and high tumor grade may need longer operation time and more extensive organ resection, resulting in increased intraoperative bleeding and a higher risk anemia after operation. Patients with low preoperative HB are less tolerant of bleeding, and even a small amount of bleeding may lead to postoperative anemia. The reconstruction and recovery of gastrointestinal function in patients with CRS + HIPEC may also be an important factor for anemia. Studies show that anemia is a prognostic factor in cancer patients (42–44), and concurrent anemia can worsen prognosis (35, 45). Wan et al. found that concurrent anemia in patients with lung, colorectal, breast, and liver cancers shortened survival compared to that in patients without anemia (46). In addition, reversing postoperative anemia in patients with late-stage large-cell neuroendocrine carcinoma can improve the quality of life and extend survival (47). Both preoperative and postoperative anemia can significantly enhance postoperative mortality rates (36, 48). In this study, we found that patients with PMP exhibiting normal Hb levels within 24h following CRS+HIPEC had higher 5-year survival rates, as well as longer median survival, than those with anemia. Furthermore, we also observed a trend of declining median survival with increasing severity of anemia.

Interestingly, postoperative anemia did not affect tumor recurrence in patients with PMP. Previous studies have shown that the degree of cytoreduction (34, 49), PCI (50–53), and tumor histological type are important prognostic factors for PMP (34, 54). In our study, postoperative CCR2–4, PCI > 20, and tumor grade 3–4 were identified as independent factors affecting patient prognosis and OS. Although the postoperative Hb level was also an independent risk factor in the normal Hb group and anemia subgroups, no significant differences were observed in the binary classification of normal versus anemic. This can be attributed to the high rate of postoperative anemia in patients with PMP, resulting in fewer patients with normal Hb levels and therefore insufficient statistical power. The severity of postoperative anemia was correlated with median 5-year OS, and patients with Hb levels of 81–90 g/L had the shortest median survival. This might be attributed to the fact that patients with Hb levels lower than 80 g/L exhibit more obvious clinical symptoms of ischemia and hypoxia and therefore receive urgent medical attention and timely blood transfusion. In contrast, those with Hb levels of 81–90 g/L lack overt clinical signs, and they are less likely to receive timely transfusion given the limited supply of allogenic blood. Consequently, ischemia and hypoxia are not effectively improved in these patients, resulting in shorter median survival. Therefore, timely blood transfusion may improve prognosis and extend the median postoperative survival of patients with PMP.

Owing to its single-center, retrospective design, this study had certain limitations. Given the high rate of postoperative anemia in patients with PMP, there were fewer patients with normal Hb levels, which necessitated an increase in the sample size to enhance statistical efficiency. Moreover, we only examined the correlation between the degree of postoperative anemia and patient prognosis. Further research is needed to assess the impact of preoperative anemia on prognosis. There are several possible reasons that may explain the poor prognosis in patients with postoperative anemia. First, the oxygen carrying capacity of patients with anemia is reduced (55), leading to insufficient oxygen supply that impairs tissue repair and slows down the healing. Second, due to the characteristics of PMP and the complexity of surgery, the reconstruction of gastrointestinal function after surgery directly affects the nutritional status of patients. Malnutrition can trigger or aggravate anemia (56, 57), weaken the immune system, and increase the risk of postoperative infection. Third, the insufficient oxygen supply caused by compensatory anemia leads to increased cardiac load, resulting in increased risk of ischemia and thrombosis (58). Finally, anemia also reduces the patient’s ability to move (59), resulting in prolonged and poor recovery after surgery.

In summary, patients with PMP are prone to develop anemia within 24h of undergoing surgery, which can significantly affect their 5-year survival rates. Furthermore, the severity of postoperative anemia is a crucial risk factor in patients with PMP. Therefore, it is essential to monitor and stratify these patients based on the severity of anemia, and assess the status of ischemia and hypoxia to identify those requiring blood component transfusion, and thus improve prognosis. Post-surgery malnutrition is an important cause of anemia in PMP patients, and the decline in immune function due to anemia increases the risk of infection and inflammation. However, we did not evaluate the association between malnutrition, inflammation, and the prognosis. We will address the prognostic relevance of the nutrition-inflammation axis in PMP patients in a follow-up study.

## Data Availability

The raw data supporting the conclusions of this article will be made available by the authors, without undue reservation.

## References

[1] Rizvi SA Syed W Shergill R . Approach to pseudomyxoma peritonei. World J gastrointestinal surg. (2018) 10:49. doi: 10.4240/wjgs.v10.i5.49, PMID: 30190782 PMC6121001

[2] Carr NJ Cecil TD Mohamed F Sobin LH Sugarbaker PH Gonzalez-Moreno S . A consensus for classification and pathologic reporting of pseudomyxoma peritonei and associated appendiceal neoplasia: the results of the Peritoneal Surface Oncology Group International (PSOGI) modified Delphi process. Am J Surg pathol. (2016) 40:14–26. doi: 10.1097/PAS.0000000000000535, PMID: 26492181

[3] Morera-Ocon FJ Navarro-Campoy C . History of pseudomyxoma peritonei from its origin to the first decades of the twenty-first century. World J Gastrointestinal Surg. (2019) 11:358. doi: 10.4240/wjgs.v11.i9.358, PMID: 31572561 PMC6766476

[4] Sommariva A Tonello M Rigotto G Lazzari N Pilati P Calabrò ML . Novel perspectives in pseudomyxoma peritonei treatment. Cancers. (2021) 13:5965. doi: 10.3390/cancers13235965, PMID: 34885075 PMC8656832

[5] Carr NJ Finch J Ilesley IC Chandrakumaran K Mohamed F Mirnezami A . Pathology and prognosis in pseudomyxoma peritonei: a review of 274 cases. J Clin pathol. (2012) 65:919–23. doi: 10.1136/jclinpath-2012-200843, PMID: 22718846

[6] Yan F Shi F Li X Yu C Lin Y Li Y . Clinicopathological characteristics of pseudomyxoma peritonei originated from ovaries. Cancer Manage Res. (2020) 12:7569–78. doi: 10.2147/CMAR.S264474, PMID: 32904568 PMC7457389

[7] Kataoka A Ito K Takemura N Inagaki F Mihara F Gohda Y . Immunohistochemical staining as supportive diagnostic tool for pseudomyxoma peritonei arising from intraductal papillary mucinous neoplasm: A report of two cases and literature review. Pancreatology. (2020) 20:1226–33. doi: 10.1016/j.pan.2020.06.008, PMID: 32768178

[8] Gohda Y Noguchi R Horie T Igari T Nakamura H Ohta Y . Pseudomyxoma peritonei of a mature ovarian teratoma caused by mismatch repair deficiency in a patient with Lynch syndrome: a case report. BMC Med Genet. (2016) 17:94. doi: 10.1186/s12881-016-0356-5, PMID: 27938333 PMC5148915

[9] Gong Y Wang X Zhu Z . Pseudomyxoma peritonei originating from transverse colon mucinous adenocarcinoma: a case report and literature review. Gastroenterol Res Practice. (2020) 2020:5826214. doi: 10.1155/2020/5826214, PMID: 32714388 PMC7354658

[10] Wambura C Jusabani A Sherman O Surani S . Pseudomyxoma pleurii and peritonei secondary to sigmoid colon adenocarcinoma: a rare clinico-pathologico-radiological presentation. Oxford Med Case Rep. (2018) 2018:omy057. doi: 10.1093/omcr/omy057, PMID: 30250743 PMC6142713

[11] Liang L Zhou N Xu H Liu D Lu Y Li F . Urachal mucinous adenocarcinoma with pseudomyxoma peritonei: a case report. Medicine. (2017) 96:e7548. doi: 10.1097/MD.0000000000007548, PMID: 28858081 PMC5585475

[12] Mittal R Chandramohan A Moran B . Pseudomyxoma peritonei: natural history and treatment. Int J Hyperthermia. (2017) 33:511–9. doi: 10.1080/02656736.2017.1310938, PMID: 28540829

[13] Baratti D Kusamura S Milione M Pietrantonio F Caporale M Guaglio M . Pseudomyxoma peritonei of extra-appendiceal origin: a comparative study. Ann Surg Oncol. (2016) 23:4222–30. doi: 10.1245/s10434-016-5350-9, PMID: 27352203

[14] Szych C Staebler A Connolly DC Wu R Cho KR Ronnett BM . Molecular genetic evidence supporting the clonality and appendiceal origin of pseudomyxoma peritonei in women. Am J pathol. (1999) 154:1849–55. doi: 10.1016/S0002-9440(10)65442-9, PMID: 10362811 PMC1866622

[15] Ronnett BM Shmookler BM Diener-West M Sugarbaker PH Kurman RJ . Immunohistochemical evidence supporting the appendiceal origin of pseudomyxoma peritonei in women. Int J Gynecological Pathol. (1997) 16:1–9. doi: 10.1097/00004347-199701000-00001, PMID: 8986525

[16] Smeenk R Van Velthuysen M Verwaal V Zoetmulder F . Appendiceal neoplasms and pseudomyxoma peritonei: a population based study. Eur J Surg Oncol (EJSO). (2008) 34:196–201. doi: 10.1016/j.ejso.2007.04.002, PMID: 17524597

[17] Blaj S Dora D Lohinai Z Herold Z Szasz AM Herzberg J . Prognostic factors in pseudomyxoma peritonei with emphasis on the predictive role of peritoneal cancer index and tumor markers. Cancers. (2023) 15:1326. doi: 10.3390/cancers15041326, PMID: 36831667 PMC9954733

[18] Patrick-Brown TDJ Carr NJ Swanson DM Larsen S Mohamed F Flatmark K . Estimating the prevalence of pseudomyxoma peritonei in Europe using a novel statistical method. Ann Surg Oncol. (2020) 28:252. doi: 10.1245/s10434-020-08655-8, PMID: 32488520 PMC7752784

[19] Dayal S Taflampas P Riss S Chandrakumaran K Cecil TD Mohamed F . Complete cytoreduction for pseudomyxoma peritonei is optimal but maximal tumor debulking may be beneficial in patients in whom complete tumor removal cannot be achieved. Dis colon rectum. (2013) 56:1366–72. doi: 10.1097/DCR.0b013e3182a62b0d, PMID: 24201390

[20] L Y X H P Z C S W W L H . Expert consensus on the treatment of peritoneal pseudomyxoma with tumor cytoreductive surgery and intraperitoneal hyperthermic perfusion chemotherapy. Natl Med J China. (2019) 99:9. doi: 10.3760/cma.j.issn.0376-2491.2019.20.003

[21] Moran B Baratti D Yan TD Kusamura S Deraco M . Consensus statement on the loco-regional treatment of appendiceal mucinous neoplasms with peritoneal dissemination (pseudomyxoma peritonei). J Surg Oncol. (2008) 98:277–82. doi: 10.1002/jso.21054, PMID: 18726894

[22] Lin J Wang C Liu J Yu Y Wang S Wen A . Prevalence and intervention of preoperative anemia in Chinese adults: a retrospective cross-sectional study based on national preoperative anemia database. EClinicalMedicine. (2021) 36:100894. doi: 10.1016/j.eclinm.2021.100894, PMID: 34041460 PMC8144738

[23] Hazen YJ Noordzij PG Gerritse BM Scohy TV Houterman S Bramer S . Preoperative anaemia and outcome after elective cardiac surgery: a Dutch national registry analysis. Br J Anaesthesia. (2022) 128:636–43. doi: 10.1016/j.bja.2021.12.016, PMID: 35031105

[24] Wischmeyer PE Carli F Evans DC Guilbert S Kozar R Pryor A . American society for enhanced recovery and perioperative quality initiative joint consensus statement on nutrition screening and therapy within a surgical enhanced recovery pathway. Anesth Analgesia. (2018) 126:1883–95. doi: 10.1213/ANE.0000000000002743, PMID: 29369092

[25] El Ghouayel M Hamidi M Mazis C Imam Z Abbad M Boutall A . Surgical outcomes in patients with preoperative anemia undergoing colectomy for colon cancer. J Surg Res. (2022) 273:218–25. doi: 10.1016/j.jss.2021.12.030, PMID: 35101682

[26] Bruns ER Borstlap WA Van Duijvendijk P van der Zaag-Loonen HJ Buskens CJ Van Munster BC . The association of preoperative anemia and the postoperative course and oncological outcome in patients undergoing rectal cancer surgery: a multicenter snapshot study. Dis Colon Rectum. (2019) 62:823–31. doi: 10.1097/DCR.0000000000001360, PMID: 31188183

[27] Urban P Mehran R Colleran R Angiolillo DJ Byrne RA Capodanno D . Defining high bleeding risk in patients undergoing percutaneous coronary intervention: a consensus document from the Academic Research Consortium for High Bleeding Risk. Eur Heart J. (2019) 40:2632–53. doi: 10.1093/eurheartj/ehz372, PMID: 31116395 PMC6736433

[28] Al-Hijji MA Gulati R Lennon RJ Bell M El Sabbagh A Park JY . Outcomes of percutaneous coronary interventions in patients with anemia presenting with acute coronary syndrome. Mayo Clinic Proc. (2018) 93:1448-1461. Elsevier. doi: 10.1016/j.mayocp.2018.03.030, PMID: 30286831

[29] Hariri E Hansra B Barringhaus KG Mohamud D Smith CS Akhter MW . Trends, predictors, and outcomes associated with 30-day hospital readmissions after percutaneous coronary intervention in a high-volume center predominantly using radial vascular access. Cardiovasc Revascularization Med. (2020) 21:1525–31. doi: 10.1016/j.carrev.2020.05.017, PMID: 32576452

[30] Cao D Mehran R Dangas G Baber U Sartori S Chandiramani R . Validation of the academic research consortium high bleeding risk definition in contemporary PCI patients. J Am Coll Cardiol. (2020) 75:2711–22. doi: 10.1016/j.jacc.2020.03.070, PMID: 32466887

[31] Li Y Yu Y Liu Y . Report on the 9 (th) international congress on peritoneal surface Malignancies. Cancer Biol Med. (2014) 11:281–4., PMID: 25610715 10.7497/j.issn.2095-3941.2014.04.008 PMC4296089

[32] Delhorme J-B Elias D Varatharajah S Benhaim L Dumont F Honoré C . Can a benefit be expected from surgical debulking of unresectable pseudomyxoma peritonei? Ann Surg Oncol. (2016) 23:1618–24., PMID: 26678404 10.1245/s10434-015-5019-9

[33] Kelly KJ . Management of appendix cancer. Clinics colon rectal surg. (2015) 28:247–55. doi: 10.1055/s-0035-1564433, PMID: 26648795 PMC4655112

[34] Ansari N Chandrakumaran K Dayal S Mohamed F Cecil T Moran B . Cytoreductive surgery and hyperthermic intraperitoneal chemotherapy in 1000 patients with perforated appendiceal epithelial tumours. Eur J Surg Oncol (EJSO). (2016) 42:1035–41. doi: 10.1016/j.ejso.2016.03.017, PMID: 27132072

[35] Narasimhan V Pham T Warrier S Craig Lynch A Michael M Tie J . Outcomes from cytoreduction and hyperthermic intraperitoneal chemotherapy for appendiceal epithelial neoplasms. ANZ J Surg. (2019) 89:1035–40. doi: 10.1111/ans.14985, PMID: 30685879

[36] Spahn DR . Anemia and patient blood management in hip and knee surgery: a systematic review of the literature. Anesthesiology. (2010) 113:482–95. doi: 10.1097/ALN.0b013e3181e08e97, PMID: 20613475

[37] Seicean A Seicean S Alan N Schiltz NK Rosenbaum BP Jones PK . Preoperative anemia and perioperative outcomes in patients who undergo elective spine surgery. Spine. (2013) 38:1331–41. doi: 10.1097/BRS.0b013e3182912c6b, PMID: 23524867

[38] Lasocki S Krauspe R Von Heymann C Mezzacasa A Chainey S Spahn DR . PREPARE: the prevalence of perioperative anaemia and need for patient blood management in elective orthopaedic surgery: a multicentre, observational study. Eur J Anaesthesiology| EJA. (2015) 32:160–7. doi: 10.1097/EJA.0000000000000202, PMID: 25564780

[39] Vochteloo AJ Borger van der Burg BL Mertens BJ Niggebrugge AH de Vries MR Tuinebreijer WE . Outcome in hip fracture patients related to anemia at admission and allogeneic blood transfusion: an analysis of 1262 surgically treated patients. BMC musculoskeletal Disord. (2011) 12:262. doi: 10.1186/1471-2474-12-262, PMID: 22104041 PMC3226448

[40] De la Garza Ramos R Goodwin CR Jain A Abu-Bonsrah N Fisher CG Bettegowda C . Development of a metastatic spinal tumor frailty index (MSTFI) using a nationwide database and its association with inpatient morbidity, mortality, and length of stay after spine surgery. World neurosurg. (2016) 95:548–55.e4. doi: 10.1016/j.wneu.2016.08.029, PMID: 27544340

[41] Sugarbaker PH . Peritonectomy procedures. Surg Oncol Clinics. (2003) 12:703–27. doi: 10.1016/S1055-3207(03)00048-6, PMID: 14567026

[42] Henry DH Langer CJ McKenzie RS Piech CT Senbetta M Schulman KL . Hematologic outcomes and blood utilization in cancer patients with chemotherapy-induced anemia (CIA) pre-and post-national coverage determination (NCD): results from a multicenter chart review. Supportive Care Cancer. (2012) 20:2089–96. doi: 10.1007/s00520-011-1318-2, PMID: 22160485

[43] Rink M Sharifi N Fritsche H-M Aziz A Miller F Kluth LA . Impact of preoperative anemia on oncologic outcomes of upper tract urothelial carcinoma treated with radical nephroureterectomy. J urol. (2014) 191:316–22. doi: 10.1016/j.juro.2013.09.010, PMID: 24036235

[44] Gierth M Mayr R Aziz A Krieger S Wullich B Pycha A . Preoperative anemia is associated with adverse outcome in patients with urothelial carcinoma of the bladder following radical cystectomy. J Cancer Res Clin Oncol. (2015) 141:1819–26. doi: 10.1007/s00432-015-1957-7, PMID: 25832016 PMC11823893

[45] Holgersson G Sandelin M Hoye E Bergström S Henriksson R Ekman S . Swedish lung cancer radiation study group: the prognostic value of anaemia, thrombocytosis and leukocytosis at time of diagnosis in patients with non-small cell lung cancer. Med Oncol. (2012) 29:3176–82. doi: 10.1007/s12032-012-0247-3, PMID: 22565809

[46] Wan S Lai Y Myers RE Li B Palazzo JP Burkart AL . Post-diagnosis hemoglobin change associates with overall survival of multiple Malignancies–results from a 14-year hospital-based cohort of lung, breast, colorectal, and liver cancers. BMC cancer. (2013) 13:340. doi: 10.1186/1471-2407-13-340, PMID: 23841898 PMC3710492

[47] Sarraf KM Belcher E Raevsky E Nicholson AG Goldstraw P Lim E . Neutrophil/lymphocyte ratio and its association with survival after complete resection in non–small cell lung cancer. J Thorac Cardiovasc surg. (2009) 137:425–8. doi: 10.1016/j.jtcvs.2008.05.046, PMID: 19185164

[48] Karkouti K Wijeysundera DN Beattie WS . Risk associated with preoperative anemia in cardiac surgery: a multicenter cohort study. Circulation. (2008) 117:478–84. doi: 10.1161/CIRCULATIONAHA.107.718353, PMID: 18172032

[49] Chua TC Moran BJ Sugarbaker PH Levine EA Glehen O Gilly FN . Early-and long-term outcome data of patients with pseudomyxoma peritonei from appendiceal origin treated by a strategy of cytoreductive surgery and hyperthermic intraperitoneal chemotherapy. J Clin Oncol. (2012) 30:2449–56. doi: 10.1200/JCO.2011.39.7166, PMID: 22614976

[50] Azzam AZ Alyahya ZA Wusaibie AAA Amin TM . Cytoreductive surgery and hyperthermic intraperitoneal chemotherapy in the management of pseudomyxoma peritonei: a single-center experience. Indian J Gastroenterol. (2017) 36:452–8. doi: 10.1007/s12664-017-0799-4, PMID: 29185227

[51] Choudry HA Pai RK Shuai Y Ramalingam L Jones HL Pingpank JF . Impact of cellularity on oncologic outcomes following cytoreductive surgery and hyperthermic intraperitoneal chemoperfusion for pseudomyxoma peritonei. Ann Surg Oncol. (2018) 25:76–82. doi: 10.1245/s10434-017-6214-7, PMID: 29110275

[52] Grotz TE Royal RE Mansfield PF Overman MJ Mann GN Robinson KA . Stratification of outcomes for mucinous appendiceal adenocarcinoma with peritoneal metastasis by histological grade. World J Gastrointestinal Oncol. (2017) 9:354. doi: 10.4251/wjgo.v9.i9.354, PMID: 28979717 PMC5605335

[53] Baratti D Kusamura S Milione M Bruno F Guaglio M Deraco M . Validation of the recent PSOGI pathological classification of pseudomyxoma peritonei in a single-center series of 265 patients treated by cytoreductive surgery and hyperthermic intraperitoneal chemotherapy. Ann Surg Oncol. (2018) 25:404–13. doi: 10.1245/s10434-017-6252-1, PMID: 29159742

[54] Narasimhan V Wilson K Britto M Warrier S Lynch AC Michael M . Outcomes following cytoreduction and HIPEC for pseudomyxoma peritonei: 10-year experience. J Gastrointestinal Surg. (2020) 24:899–906. doi: 10.1007/s11605-019-04239-4, PMID: 31090036

[55] Spinelli E Bartlett RH . Anemia and transfusion in critical care: physiology and management. J Intensive Care Med. (2016) 31:295–306. doi: 10.1177/0885066615571901, PMID: 25693602

[56] Seo HS Na Y Jung J . Analysis of the occurrence of diseases following gastrectomy for early gastric cancer: a nationwide claims study. J Gastric Cancer. (2021) 21:279. doi: 10.5230/jgc.2021.21.e29, PMID: 34691812 PMC8505120

[57] Ganz T . Anemia of inflammation. New Engl J Med. (2019) 381:1148–57. doi: 10.1056/NEJMra1804281, PMID: 31532961

[58] Fabjan TH Penko M Hojs R . Anemia on admission and long-term mortality risk in patients with acute ischemic stroke. Adv Clin Exp Med. (2019) 28:1419–24. doi: 10.17219/acem/104540, PMID: 31538415

[59] González Marcos E González García E González-Santos J González-Bernal JJ del Pilar Martín-Rodríguez A Santamaría-Peláez M . Determinants of lack of recovery from dependency and walking ability six months after hip fracture in a population of people aged 65 years and over. J Clin Med. (2022) 11:4467. doi: 10.3390/jcm11154467, PMID: 35956084 PMC9369508

